# Development of a Low-Cost 6 DOF Brick Tracking System for Use in Advanced Gas-Cooled Reactor Model Tests

**DOI:** 10.3390/s22031110

**Published:** 2022-02-01

**Authors:** Paolo Olson, Adam J. Crewe, Tansu Gokce, Tony Horseman, Rory E. White

**Affiliations:** 1 Plant Systems and Safety, Atkins, Bristol BS32 4RZ, UK; paolo.olson@atkinsglobal.com; 2 Earthquake and Geotechnical Engineering Group, Faculty of Engineering, Bristol BS8 1TR, UK; tansu.gokce@bristol.ac.uk (T.G.); tony.horseman@bristol.ac.uk (T.H.); rory.white@bristol.ac.uk (R.E.W.)

**Keywords:** monocular camera, 6 DOF, pose estimation, sensor fusions, time synchronisation

## Abstract

This paper presents the design of a low-cost, compact instrumentation system to enable six degree of freedom motion tracking of acetal bricks within an experimental model of a cracked Advanced Gas-Cooled Reactor (AGR) core. The system comprises optical and inertial sensors and capitalises on the advantages offered by data fusion techniques. The optical system tracks LED indicators, allowing a brick to be accurately located even in cluttered images. The LED positions are identified using a geometrical correspondence algorithm, which was optimised to be computationally efficient for shallow movements, and complex camera distortions are corrected using a versatile Incident Ray-Tracking calibration. Then, a Perspective-Ray-based Scaled Orthographic projection with Iteration (PRSOI) algorithm is applied to each LED position to determine the six degree of freedom pose. Results from experiments show that the system achieves a low Root Mean Squared (RMS) error of 0.2296 mm in x, 0.3943 mm in y, and 0.0703 mm in z. Although providing an accurate measurement solution, the optical tracking system has a low sample rate and requires the line of sight to be maintained throughout each test. To increase the robustness, accuracy, and sampling frequency of the system, the optical system can be augmented with an Inertial Measurement Unit (IMU). This paper presents a method to integrate the optical system and IMU data by accurately timestamping data from each set of sensors and aligning the two coordinate axes. Once miniaturised, the developed system will be used to track smaller components within the AGR models that cannot be tracked with current instrumentation, expanding reactor core modelling capabilities.

## 1. Introduction

**Figure 1:**
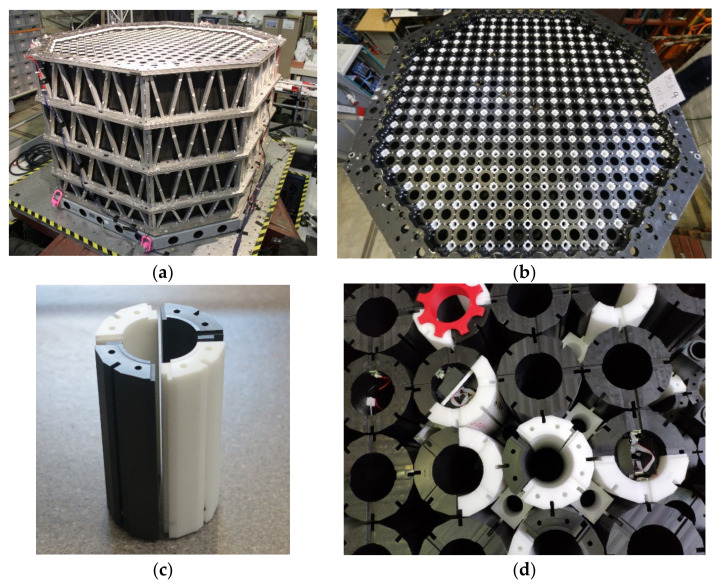
Multi-Layer Array rig and bricks: (**a**) Side view of MLA; (**b**) Top view of MLA; (**c**): A Quadruply Cracked Brick (QCB); (**d**) Detail of partially built MLA with intact, DCB, TCB, and QCB bricks, QCB brick held closed by red “spider” ring.

Within the UK, several Advanced Gas-Cooled Reactor (AGR) cores are currently approaching the end of their design life [1]. Each AGR core [2] is constructed using graphite bricks interconnected by a keying system. As the graphite bricks age, prolonged irradiation can result in material degradation and cracking. Understanding the effect of brick degradation is an important factor to maintain safe operations. The safety case for each AGR facility includes provision for safe operation during a seismic event with a 10^−4^ probability of occurrence per annum, specifically the ability to shut down, hold down, and cool the reactor core. In collaboration with EDF and SNC-Lavalin’s Atkins business’, the University of Bristol developed the Multi-Layer Array (MLA) [3], which is a quarter-sized model of an AGR core. During experiments, the MLA (Figure 1a,b) is installed on the University of Bristol shaking table to investigate the dynamic response of the array subjected to seismic events. Results obtained from MLA experiments provide validation and verification data for finite element models, which, in turn, enable detailed analysis of the AGR core components.

To simulate the effect of cracked bricks within the AGR core, components within the quarter-sized array were modified to replicate cracked bricks. Of particular interest in this study are the lattice bricks, which model the AGR fuel bricks. Four different lattice brick types can be installed during an experiment: uncracked bricks, bricks that are cracked in two and referred to as Doubly Cracked Bricks (DCB), and bricks cracked in four and referred to as Quadruply Cracked Bricks (QCB) (see the black and white cylinders in Figure 1c,d). It is also possible to combine two QCB components with one DCB component to make a Triply Cracked Brick (TCB). When the core experiences a seismic event, the presence of cracked bricks increases the dynamic displacement of the core, disrupting the uniform geometry with potential ramifications for fuel cooling and control rod insertion. While instrumentation had been developed to track the relative motion of the uncracked bricks and DCBs [4,5,6], the methods used for these bricks were not viable for use with TCBs and QCBs. The particular challenges with measuring the motion between the four QCB components are the potential for relatively large six degree of freedom (DOF) motion between each quarter, very limited space for sensors, and a need for non-contact sensors to avoid the instrumentation interfering with the brick dynamics. This paper describes the design and development of cheap, compact instrumentation, which will satisfy these design constraints and is also capable of achieving sub-millimetre and sub-degree accuracy in all six DOF between the QCB components. Developing this compact and accurate instrumentation was essential, as it is necessary to show that the relative motions of the TCB and QCB bricks in the MLA during testing agrees with that produced by FE modelling of the array, thus helping to validate the modelling approach being used for the reactor safety cases.

The paper starts by outlining the instrumentation concept before discussing the selection of an appropriate optical tracking algorithm. The physical system, which uses cheap, readily available components, is then described, which is followed by a detailed description of the optical tracking method developed, which produces sub-millimetre and sub-degree accuracy. Timestamping and aligning the axes of the optical and inertial data and the future development is finally discussed, showing that it is possible to develop a cheap but compact instrumentation system that will allow accurate monitoring of the relative motions of the TCB and QCB bricks during MLA testing.

## 2. Instrumentation Concept

Instrumentation of the QCBs involves several design constraints, which increase the complexity of the problem. Sensors must be small enough to be housed within the brick itself, and in the case of a lattice brick, this corresponds to a cylindrical cavity with an internal radius of 32.900 mm, external radius of 57.375 mm, and height of 208.750 mm. This meant that the system would have to use small cameras with short focal lengths and wide angled lenses. Therefore, the developed system would have to be able to compensate for image distortion. The MLA comprises 585 columns of stacked bricks, 284 of which are columns of lattice bricks. There are seven lattice bricks per lattice column, leaving no clear line of sight to the majority of the bricks in the array (see Figure 1a). Any instrumentation design must make use of non-contact sensors to ensure that the measurement system does not interfere with the dynamic behaviour of the bricks. Acquired data must be time synchronised so that the local displacement measurements can be directly compared to the global motion of the array and the seismic input profile. Data must also be sampled at sufficiently high sampling rates to ensure that all high-frequency motion is captured by the system. Finally, the instrumentation should be as low-cost as possible so that it is economically viable to instrument up to 400 bricks within the array.

Inertial and optical tracking systems were identified as potentially suitable non-contact sensors that could meet all design requirements. Inertial tracking is the high frequency sensor fusion of accelerometers, gyroscopes and, in some cases, magnetometers, to accurately define the six DOF movement of an object. Accelerometers and gyroscopes measure linear acceleration and angular velocity, respectively. To calculate the orientation and displacement of the object, the angular acceleration and linear acceleration are integrated twice with respect to time. Unfortunately, both gyroscopes and accelerometers are susceptible to instabilities that are also integrated over time, causing the calculated pose to drift. To reduce these issues, magnetometers can be used to ameliorate the drift in measurements by providing a fixed orientation and linear acceleration reference to the earth’s magnetic field.

Optical tracking systems provide accurate measurement of position and orientation without drift in the recorded data, but they have slow update rates, which fail to capture high-frequency motion. Optical systems can also be unreliable due to occlusions or motion blur. To develop a design using either inertial or optical sensors that is compact, accurate, and with a high sample frequency, specialised instrumentation is required. Such high-performance equipment is typically expensive and was beyond the budget of this project when considering the number of bricks that it was desirable to track. As an alternative approach, low-cost cameras and low-cost IMUs can be sensor-fused to exploit the advantages of both technologies. Sensor fusion uses the accurate data from the optical tracking system to correct the drift in the IMU data, producing a system that yields high-accuracy measurements at high sample rates.

## 3. Selection of Optical Tracking Algorithm

All optical motion-tracking systems use feature identification and tracking of an object in a 2D image. Lattice bricks have a uniform bore and in low light conditions, small markers will need to be placed on the brick to identify points of reference. Many different possible markers could be used, such as retroreflective markers, Secchi markers [7], or LEDs [8]. LED markers, in particular, have been used inexpensively and effectively in many applications (e.g., He et al. [7], Rambach et al. [9], and Savkin et al. [10]) and can be quickly identified in an image through the use of thresholding, segmentation, and morphological operations.

Once the markers have been identified in an image, an optical tracking algorithm is applied to the computed 2D coordinates. Only monocular cameras were considered for the QCB instrumentation owing to spatial and cost constraints so the optical tracking algorithms considered could not require input from multiple cameras. Two of the most widely used optical tracking algorithms are the Perspective n-Point (PnP) [11] algorithm and the Pose from Orthography and Scaling with Iteration (POSIT) [12] algorithm. These algorithms have been adapted to a wide range of problems and have been extended to provide enhanced capability, which better suits the application at hand.

The PnP has many variations, including the Unified PnP (UPnP) [13], the Lu, Hager, and Mjolsness method (LHM) [14], and the Maximum Likelihood PnP (MLPnP) [15]. PnP algorithms can be split into two categories: iterative and non-iterative. Many non-iterative PnP algorithms are suitable for real-time applications and have sub-centimetre accuracy. Iterative algorithms are slower but have higher accuracy when they converge correctly. The MLA experiments do not require real-time analysis, as all data could be post-processed; thus, computational efficiency is of less importance than accuracy, and iterative algorithms are appropriate. However, all PnP algorithms require a camera calibration to determine the focal length and to correct distortions in the camera lens. Additionally, the PnP algorithms require a large number of markers or points [13,15] before they produce sub-millimetre and sub-degree accuracy, which would be difficult in a space-restricted environment, as is the case here.

An alternative, the POSIT algorithm [12], requires far fewer points to give sub-millimetre and sub-degree accuracy. The method applies scaled orthographic projection and uses the object’s geometrical characteristics to find the rotation and translation matrix between the object and image coordinate systems. Iterating the process achieves better scaling of the orthographic projections, improving accuracy. The algorithm has been shown to converge globally without requiring an initial guess and is highly accurate.

The difficulty in applying a POSIT algorithm in this case is the requirement for a priori knowledge of the correspondence between 2D image points and 3D object points. In addition, the accuracy of the algorithm deteriorates significantly as points become planar. The success of the POSIT algorithm led to the development of other iterative algorithms such as the SoftPOSIT algorithm [16]. The SoftPOSIT algorithm finds the 2D image and 3D object correspondence by combining the POSIT with the softassign algorithm [17], which finds correspondence using an iterative spatial mapping and Procrustes re-scaling. This resulted in the algorithm being much more robust to cluttered images, occlusions, and image noise while being more computationally efficient than the POSIT algorithm.

Similarly, deep learning methods have been shown to be accurate, stable, and achieve real-time performance. Zheng et al. [18] and Carrillo and Vamvoudakis [19] in particular have shown that deep learning is particularly robust even in noisy environments and with significant interference. However, although Garon and Lalonde [20] have shown deep-learning to be very robust to occlusions and a real-time algorithm for image processing, these methods do not provide sub-degree or sub-millimetre accuracy, making deep-learning algorithms unsuitable for this application.

Due to the higher levels of accuracy, the algorithm ultimately selected for this application was the Perspective-Ray-based Scaled Orthographic projection with Iteration algorithm with an Incident Ray Tracking calibration (PRSOI-IRT) [21]. The PRSOI-IRT uses orthographical projection to map object feature points onto two highly calibrated planes. Then, the code refines the projection of the points to find to the object pose in the form of a translation and rotation matrix. The algorithm is more accurate than either the softPOSIT, POSIT, or PnP algorithms for fewer points [21]. Furthermore, the Incident Ray Tracking (IRT) calibration, unlike the PnP algorithm, is included in the pose determination, allowing the algorithm to correct the distortion of any monocular camera. This is particularly convenient given that this is the first version of this instrumentation design, as it allows the instrumentation to be versatile if upgrades need to be made to the camera in the future.

Softassign [17] could be incorporated into the PRSOI-IRT, similar to the SoftPOSIT method, to find the 2D–3D correspondence but would result in a more computationally expensive and complex algorithm. Alternatively, Tjaden et al. [8] showed that correspondence can be robustly and efficiently found when fixing markers linearly. The markers are arranged in a cross-like pattern made up of three and five linear markers. Then, the linear markers are identified by fitting a line to each possible combination of three and five markers, evaluating the angle of the fitted line and determining the distance of the furthest outlier from the line. These principles were applied in this paper to efficiently find the 2D-3D correspondence (see Section 5.3).

## 4. System Description

**Figure 2:**
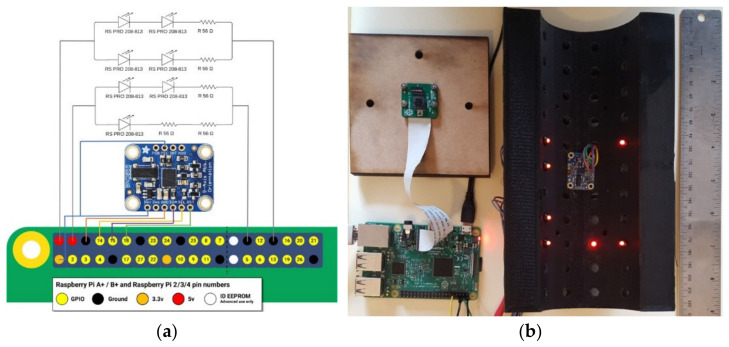
System configuration: (**a**) Wiring; (**b**) Full System.

Based on the design requirements outlined in Section 2, a Raspberry Pi 3B was used for data acquisition. As a relatively compact, single board computer, the Raspberry Pi 3B offers a range of input ports and a powerful processor that provides versatility should instrumentation requirements change in the future. A Raspberry Pi v2 camera module and seven RS PRO Red Indicators comprised the optical system components. The camera was connected to the Raspberry Pi via the Camera Serial Interface (CSI) to produce 1920 × 1080 8 MP video at 30 Frames Per Second (FPS). Images were stored on a Toshiba Exceria M303 64GB microSDXC. This camera was selected for its wide 62.2° × 48.8° field of view (FOV), allowing it to capture a large area of the brick when in close proximity, its small geometrical size (25 × 23.86 × 9 mm), and low cost. However, the Raspberry Pi v2 camera has a fixed focus range from 1 m to infinity. To reduce this to a suitable range, the lens was unscrewed by 180°, resulting in images focusing from 10 cm away. The 6 mm RS PRO Red Indicators were selected based on their low cost, rigid fixing, and small size. An Adafruit BNO055 Absolute Orientation Inertial Measurement Unit (IMU) was selected for its ability to combine a triaxial 16-bit gyroscope, a triaxial 14-bit accelerometer, and a triaxial geomagnetic sensor via a 32-bit microcontroller into a tiny 26.67 × 20.32 × 1.18 mm board. Both indicators and the IMU were connected to the Pi via the General Purpose Input/Output (GPIO) ports (see Figure 2a).

A prototype lattice brick was 3D printed from PolyLactic Acid (PLA) using an Ultimaker 2+ with a uniform pattern of 42 holes to insert LEDs and a small flat surface at the centre of the brick where the IMU was installed, as per Figure 2b. The system was implemented on a DCB rather than QCB so that it was possible to investigate several possible LED configurations and enable comparison against the motion-tracking instrumentation currently available in the lab. In this paper, the configuration of LED installation was set to 118° of the brick, which most closely simulated an arrangement that would work in a quarter-brick layout. When the system is installed in QCB bricks, the LEDs will be placed closer together (therefore occupying a smaller angle), which just changes the scaling of the LED pattern in the camera view. If necessary, this can be compensated for by using a camera lens with a narrower field of view. The most important issue in the installation of the LEDs is that the LEDs are not all coplanar, as coplanar points can lead to inaccuracies in the pose estimation

The entire system was controlled by a PC (Lenovo Thinkpad E470 made by Lenovo PC HK Limited, Hong Kong, China) via an Ethernet connection to the Raspberry Pi. At the time of publication, the cost of a Raspberry Pi 3B was £34, the IMU was £7.80, the camera was £24.40, the LED indicators were £1.98 each, and the microSD card was £15, resulting in a full system cost of £95.

## 5. Optical Tracking Methodology

**Figure 3:**
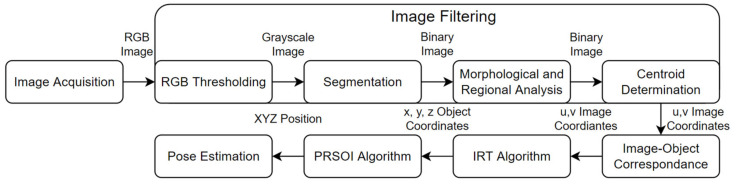
Image processing summary.

This section outlines how a 2D RGB image can be converted to a 3D pose relative to the camera. In summary, video footage of the brick is broken down into individual frames, as detailed in Section 5.1. Image Filtering first isolates and then identifies LEDs in an image, as described in Section 5.2 and Section 5.3. Then, the IRT algorithm uses rectilinear propagation to correct lens distortions and project the u, v LED image coordinates onto two virtual z-planes with real x and y positions relative to the camera, as presented in Section 5.4. Then, using the fixed and known position of the LEDs, the PRSOI algorithm can iteratively compute the six DOF pose of the brick relative to the camera, as per Section 5.5. The optical tracking methodology is summarised in Figure 3. All image processing was implemented in MATLAB R2019b.

### 5.1. Image Acquisition

The existing MLA data acquisition systems are time synchronised using a precise fixed-frequency square wave [3]. When the instrumentation detects a rising edge, a reading is taken, and the data are recorded. To simulate the MLA trigger, a Farnell FG3 Function Generator was connected to the Raspberry Pi via the GPIO pins, and the Raspberry Pi was programmed to capture a single image frame every time a rising edge was detected, indexing each image by a unique filename. This limited the frame acquisition to 0.37 FPS. To significantly improve the image acquisition rate, the Raspberry Pi was programmed to capture a 15 s video once the first rising edge was detected and ignoring all future rising edges. Then, the video can be converted into image frames. This method enables higher frame rates to be achieved; however, given that the camera is not designed for high-precision applications, it is possible that inconsistent frame rates could create inaccuracies in the frame timestamps. Issues related to accurate timestamping of images are investigated further in Section 7.

When selecting a suitable frame rate for the application at hand, a compromise must be found between the resolution of the video and the frame rate. Higher-resolution video results in more intensive image processing requirements per image and limits the achievable frame rate. In this case, the accuracy of the optical system was prioritised over the sampling frequency, and the maximum available resolution of 1920 × 1080 pixels was chosen. This resulted in a frame rate of 30 FPS, which could be improved by installing a more expensive camera.

### 5.2. Image Filtering

Image filtering aims to identify and isolate markers from an image without affecting the shape, area, or position of the markers. The image processing approach adopted here consisted of thresholding, segmentation, and morphological operations. The threshold applied filters to each RGB colour from 100 to 255, removing the darker colours of the image and producing an 8-bit RGB image. The threshold was optimised to remove as much of the darker parts of the images as possible without removing any of the external LED pixels, which could affect the accuracy of the pose estimation. Then, the image was converted to greyscale, giving the RGB colours a value from 0 to 255, with 0 being black and 255 being white. Segmentation was applied, partitioning the image into binary regions by analysing colour discontinuities between pixels. Then, the segmentation applied a foreground polarity threshold, filtering pixels darker than 200 and further isolating the LED positions in the image.

Then, morphological operations were applied to the remaining regions of interest in the image to remove regions with unsuitable eccentricity, radius, or area. Lastly, all identified regions which were cropped by the borders of the image were removed due to the centre of mass of these regions being skewed inwards towards the centre of the image, resulting in the pose of the brick being incorrectly calculated. Then, the centroid of each region was evaluated by the finding the *u* and *v* vector coordinates of each pixel within the region of interest and calculating the mean value.

**Figure 4:**
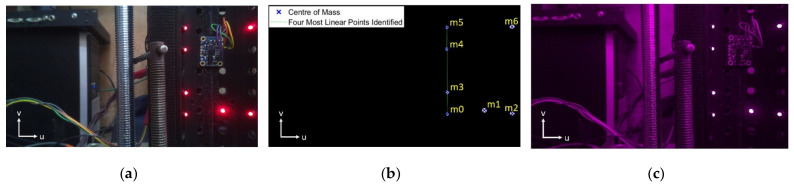
Image filtering and 2D–3D Correspondence; (**a**) Unprocessed image; (**b**) Segmented image with applied morphological operations and correspondence (see Section 5.3); (**c**) Difference between the processed and unprocessed image.

Figure 4 shows the initial and final image showing that the image processing accurately and robustly identifies the LEDs, even in cluttered images, finding the centroid of the LED with very little noise.

### 5.3. 2D–3D Image Correspondence and LED Position Determination

The PRSOI algorithm requires the correspondence between the 2D image and 3D object points to be determined. This study outlines a new, simple, computationally efficient correspondence algorithm. While other methods, such as that given in [22], exist to determine the correspondence for coplanar points using different-coloured LEDs, this method utilises the collinearity of points and the relative position of the collinear points to other LEDs to determine the 2D–3D correspondence. The algorithm was developed and tested, proving to be simple, stable, and efficient.

The 2D–3D correspondence is found by locating the four linear, coplanar LEDs (*m*_0_, *m*_3_, *m*_4_, and *m*_5_), allowing them to be quickly isolated from the other three LEDS. To find the four linear points, the algorithm comprehensively tests each of the 35 LED combinations ($C_{3}^{7}$) to find the optimal solution. To verify that the four points are collinear, one point is randomly selected from the group of seven as the reference position and termed Point A. The angle (***θ***) of each point (***u***,***v***) relative to Point A (*u_A_*, *v_A_*) can be computed using Equation (1). Note that ***θ***, ***v***, and ***u*** are shown in bold (indicating they are vectors) as the algorithm is vectorised to calculate the angle from Point A to each point more efficiently.

(1) $$\theta =\tan^{-1}\left(\frac{v-v_{A}}{u-u_{A}}\;\right)$$

**Figure 5:**
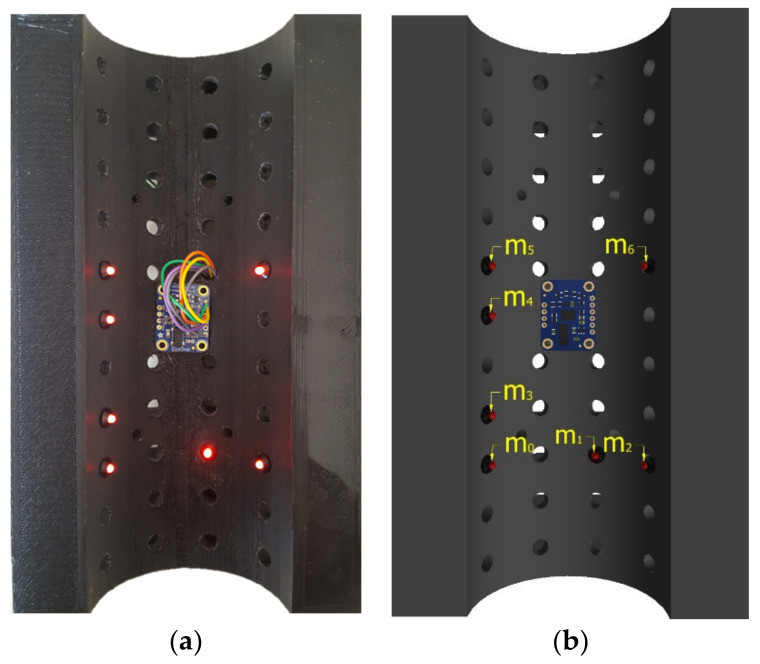
Comparison between CAD and actual brick; (**a**) Actual brick; (**b**) CAD brick.

The difference between each calculated angle is evaluated, and if the difference in angle between each point is less than 1° or larger than 179°, then the points are considered collinear. If four points do not meet this criterion, a different point is taken as Point A until a suitable Point A is found. The correspondence determination is optimised by sorting the points in ascending order by their u-coordinate. As the linear points are located on the left-hand side of the brick, sorting the points in this manner minimises the number of iterations required to find Point A when testing all 35 possible combinations. Figure 5 shows the LED positions as installed on the prototype DCB.

Once the four coplanar LEDs have been identified, the algorithm then determines the correspondence of the remaining three LEDs. To achieve this, the Chebyshev distance is evaluated by calculating the maximum differences between either of the u and v pixel coordinates of *m*_1_, *m*_2_, and *m*_6_. The correspondence of *m*_6_ is the first to be identified, as it has the greatest distance from both *m*_1_ and *m*_2_. By comparing the distance between the remaining two points and each of the coplanar coordinates, *m*_1_ and *m*_2_ can be identified as *m*_1_ is closer in position to *m*_0_, *m*_3_, *m*_4_, and *m*_5_. Although *m*_0_, *m*_3_, *m*_4_, and *m*_5_ have been identified as the collinear points, the correspondence of each is yet to be determined. To achieve this, *m*_5_ is identified as the point furthest from *m*_1_ and *m*_2_. Then, the relative distances between *m*_0_, *m*_3_, and *m*_4_ are used to discriminate between each point. As the brick is not expected to rotate more than 30°, the robustness of the algorithm was evaluated for a 100 different brick angles, varying from −45° to 45° in roll, pitch, and yaw. Correspondence was found correctly in each frame in an average of 0.0026 s on a PC (Lenovo Thinkpad E470), making the algorithm computationally efficient and robust. This method worked well when processing the greyscale images produced from the single-colour LEDS. However, it may be possible to improve the method further if multiple different colour LEDS are used and by processing the colour images in a similar way to that done by Kyriakoulis and Gasteratos [22].

To determine the pose of the brick, the 2D LED image coordinates must be compared with the LED’s relative 3D world coordinates. The 3D relative position of the LEDs was determined through the development of an Autodesk Inventor CAD model of the brick and LEDs. Such an approach was possible because of the tolerances associated with the fabrication of the brick and the LEDs. The Ultimaker 2+ has a resolution of 12.5 µm in the x- and y-directions and a resolution of 5 µm in the z-direction. The LEDs were constructed with a tolerance of 0.4 mm or less. The CAD model of the brick and installed LEDs enabled accurate measurement of the distance between the LEDs using the in-built functionality of Autodesk Inventor. The measurements were computed from the centre of volume of the LED hemispheres to ensure the best correspondence when the 3D hemispheres were mapped to the 2D LED images.

### 5.4. IRT Calibration Algorithm

To understand the PRSOI-IRT algorithm, it is important to understand the notation. Superscripts indicate the z-position of a point, subscripts indicate whether it is a non-origin point, *t*, or the origin point, o (see Section 5.5), and a bold variable indicates a matrix or vector rather than a scalar.

**Figure 6:**
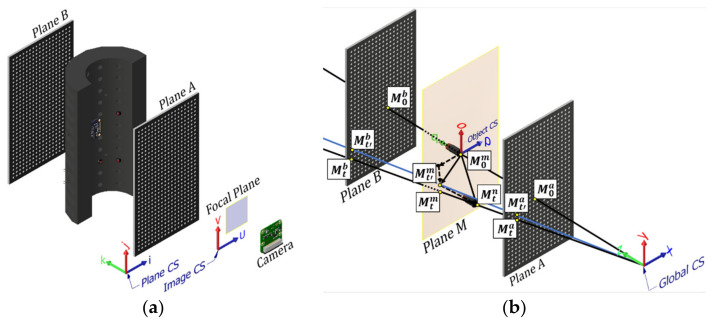
PRSOI-IRT experimental system with the two calibration boards: (**a**) IRT calibration and defined coordinate systems (**b**) IRT problem description [23].

The IRT model [23] is applied by creating two planes, Plane A and Plane B (see Figure 6a), and computing the mapping between the local u, v camera coordinate system, and x, y, and z global coordinate systems. To apply this mapping, it is not necessary to compute the camera parameters but simply define a function that maps between the two coordinate systems. This “black box” approach makes the IRT calibration highly versatile, allowing it to capture complex distortions in any monocular camera. The mapping is found by placing a plane of calibration targets with known, fixed x and y markers at the two expected z-limits of the brick’s movements. These planes of calibration points will be denoted Plane A and B. As the calibration targets are perpendicular to the camera, Plane A and B can be summarised by the user-selected z-limits of the brick’s movement from the camera, which are denoted *a* and *b*. The calibration markers should be densely distributed to ensure that the calibration captures any distortion from the lens. Then, images can be taken of the calibration target, and the u, v coordinates of the markers can be computed. The mapping between the camera coordinate system and the global coordinate system is parameterised by a non-linear polynomial of order *q*, which is described by Equations (2) and (3). Note the powers and multiplication are performed elementwise. Elementwise multiplication is denoted by “∗”, the cross product by “×” and the dot product by “·”.

(2) $$x_{t}=\overset{q}{\underset{r=0}{\sum }}\overset{q-r}{\underset{s=0}{\sum }}C_{rs}{\left(u_{t}\right)}^{r}*{\left(v_{t}\right)}^{s}$$

(3) $$y_{t}=\overset{q}{\underset{r=0}{\sum }}\overset{q-r}{\underset{s=0}{\sum }}D_{rs}{\left(u_{t}\right)}^{r}*{\left(v_{t}\right)}^{s}$$

The *C_rs_* and *D_rs_* coefficients create a polynomial function of the *q*th order that projects the LED coordinates, which are on the image plane (i.e., a plane at the focal length of the camera) to Plane A and Plane B, where each plane has its corresponding *C_rs_* and *D_rs_* coefficient. The mapping coefficients apply the transformation from the image coordinate system to the global coordinate system. This polynomial function that maps the coordinates is “black box” and is not required to know the distance of Plane A and B from the camera. The distance of Plane A and B from the camera is required for the PRSOI part of the algorithm (not the IRT algorithm), denoted by *a* and *b*, which iteratively compute the pose of the brick through approximating and refining a ray-based projection (see Section 5.5). Note that $u_{t}$**,** $v_{t}$**,** $x_{t}$**,** and $y_{t}$ are bold, as it is assumed that there is more than one calibration point.

### 5.5. PRSOI Algorithm

The PRSOI algorithm calculates the pose of the brick by solving a linear system of equations which approximate and refines a ray-based protection. For a full explanation, please refer to Guo et al. [23] and Sun et al. [21]. In summary, there are four coordinate systems: the image coordinate system (u, v) (see Figure 4), the object coordinate system (o, p, q), the plane coordinate system (i, j, k), and the global coordinate system (x, y, z) (see Figure 6). The plane and global coordinate systems are defined so that k and z are parallel to the principal axis of the camera, and (i, j) and (x, y) are parallel to the u and v axes of the image coordinate system (i.e., the global coordinate system is fixed to the camera). The plane coordinate system is centred around each plane, and the world coordinate system is centred around the camera.

The IRT calibration maps the LED coordinates from the image plane (u, v: Image coordinate system) to virtual Plane A and B (i, j, k: Plane coordinate system), as shown in Section 5.4. The object coordinate system is fixed to the brick and centred around an LED of choice known as the origin LED. To determine the pose of the brick, the relative position of the non-origin LEDs must be known relative to the origin LED (as detailed in Section 5.3) in the o, p, q coordinate system.

To determine the pose of the brick, the PRSOI algorithm must calculate the rotation matrix, *R*, and translation matrix *T* that maps from the camera to the LEDs. *R* maps from the plane coordinate system (i, j, k) to the object coordinate system: o, p, and q. The rotation matrix (i, j, k) will depend based on how the o, p, q coordinate system is defined and on the rotational position of the brick. To calculate the rotation matrix, the algorithm calculates the value of the plane unit vectors, *i*, *j*, and *k*, in the object coordinate system, as shown in Equation (4). As it is possible to compute *k* by simply taking the cross product of *i* and *j*, only vectors *i* and *j* are required to calculate the rotation matrix.

(4) $$R=\left[\begin{array}{c} i\\ j\\ k \end{array}\right]\;$$

The PRSOI must also determine the translation matrix, *T*, which is defined as the displacement from the global coordinate system to the object coordinate system/origin LED. The algorithm formulates the z-position of the non-origin LEDs as the distance from the origin LED, found on Plane M, plus (towards Plane B) or minus (towards Plane A) a distance denoted $\epsilon_{t}$. If the z-position of each LED is known, the x and y position can be calculated, as the x and y position of the LEDs is known at Plane A and B as calculated in Section 5.4. The calculation simply takes the ratio of the distance from Plane A to Plane M and Plane A to Plane B and the difference in the x-coordinate from Plane A and Plane B to calculate the relative x and y position of each LED (see Equations (25) and (26)). However, the z-position of both the origin LED and all other LEDs is initially unknown.

By assuming $\epsilon_{t}$ is zero, the distance to from the camera to the origin LED and the *i*, *j* and *k* unit vectors can be estimated by applying the linear system known as the Perspective Ray-based Scaled Orthographic projection (PRSO). Once *i*, *j*, and *k* are known, a more exact value of $\epsilon_{t}$ can be calculated, and the equations can be iterated to refine the values until the algorithm converges towards values that correspond to the pose of the LEDs. Hence, the algorithm is called PRSOI (PRSO with Iterations).

#### 5.5.1. Implementation of Algorithm

This section outlines how the PRSOI algorithm is implemented without delving into the full vector mathematics and explanation outlined in Sun et al. [21] and Guo et al. [23].

The notation of the algorithm can be described as follows. $M_{o}^{m}$ is the origin LED, and $M_{t}^{n}$ is a non-origin LED that is a z-distance $\epsilon_{t}\;$ from Plane M and a z-distance $n_{t}$ from the camera plane. When the calibration is undertaken in Section 5.4, $M_{o}^{m}$ and $M_{t}^{m}$ are projected onto Plane A to create $M_{0}^{a}$ and $M_{t}^{a}$ and onto Plane B to create $M_{0}^{b}$ and $M_{t}^{b}$**.** $M_{t}^{m}$ is the non-origin point rectilinearly propagated onto Plane M, and $M_{t’}^{m}$ is the non-origin point orthogonally projected onto Plane M. $M_{t’}^{a}$ and $M_{t’}^{b}$ are when $M_{t’}^{m}$ is rectilinearly propagated to Plane A and B. Each of these points, $M_{\left[\;\;\;\right]}^{\left[\;\;\;\right]}$**,** has an x, y, and z coordinate, which are denoted $x_{\left[\;\;\;\right]}^{\left[\;\;\;\right]}$**,** $y_{\left[\;\;\;\right]}^{\left[\;\;\;\right]}$, and $z_{\left[\;\;\;\right]}^{\left[\;\;\;\right]}$**.**

$\vec{M_{o}^{m}M_{t}^{n}}$ is described by adding two vectors together: one along Plane M, $\vec{M_{o}^{m}M_{t’}^{m}}$, and one orthogonal to the Plane M, $\vec{M_{t’}^{m}M_{t}^{n}}$ (see Equation (5)). To map the plane coordinate system onto the object coordinate system, the dot product of the vector from the origin LED to the non-origin LED must be taken relative to the i or j axes. Taking the dot product of $\vec{M_{o}^{m}M_{t}^{n}}$ with respect to *i* or *j* gives $\vec{M_{o}^{m}M_{t’}^{m}}$ as $\vec{M_{t’}^{m}M_{t}^{n}}$ is orthogonal to both the i and j axes.

(5) $$\vec{M_{o}^{m}M_{t}^{n}}=\vec{M_{o}^{m}M_{t’}^{m}}+\vec{M_{t’}^{m}M_{t}^{n}}$$

The location of point $M_{t’}^{m}$ is unknown, but we know that $x_{t’}^{m}=x_{t}^{n}$ and $y_{t’}^{m}=y_{t}^{n}$. Using these principals, it possible to express $x_{t^{\prime }}^{a}$ and $y_{t^{\prime }}^{a}$ using the proportional vector relationship between Planes A, B, and M; see Equations (6) and (7), where *m*, *a*, and *b* are the z-distance of Planes M, A, and B from the camera (where *a* and *b* were selected in Section 5.4).$\;\epsilon_{t}$ is the z-distance between point $M_{t}^{n}$ and $M_{t}^{m}$ (as shown in Equations (22) and (23)). Note the x and y coordinates of the non-origin points are denoted as vectors as it is assumed throughout this paper that there is more than one non-origin point and the algorithm can be vectorised for efficiency. Meanwhile, the origin point coordinates are scalar, as there is only one origin point.

(6) $$x_{t^{\prime }}^{a}=x_{t}^{a}+\frac{\left(x_{t}^{b}-x_{t}^{a}\right)*\left(x_{t}^{a}-x_{o}^{a}\right)}{\left(b-m\right)\left(x_{t}^{a}-x_{o}^{a}\right)+\left(m-a\right)\left(x_{t}^{b}-x_{o}^{b}\right)}*\epsilon_{t}$$

(7) $$y_{t^{\prime }}^{a}=y_{t}^{a}+\frac{\left(y_{t}^{b}-y_{t}^{a}\right)*\left(y_{t}^{a}-y_{o}^{a}\right)}{\left(b-m\right)\left(y_{t}^{a}-y_{o}^{a}\right)+\left(m-a\right)\left(y_{t}^{b}-y_{o}^{b}\right)}*\epsilon_{t}$$

Therefore, the dot product of $\vec{M_{o}^{m}M_{t}^{n}}$ with respect to the i and j axis can be computed as below where $M_{o}^{m}M_{t}^{n}$ is projected onto Plane M to produce vector ***I*** and ***J*,** where ***I*** and ***J*** are unknowns, which are defined, so Equations (8) and (9) hold.

(8) $$\vec{M_{o}^{m}M_{t}^{n}}\cdot I=x_{t^{\prime }}^{a}-x_{o}^{a}=x_{t}^{a}-x_{o}^{a}+\frac{\left(x_{t}^{b}-x_{t}^{a}\right)*\left(x_{t}^{a}-x_{o}^{a}\right)}{\left(b-m\right)\left(x_{t}^{a}-x_{o}^{a}\right)+\left(m-a\right)\left(x_{t}^{b}-x_{o}^{b}\right)}*\epsilon_{t}$$

(9) $$\vec{M_{o}^{m}M_{t}^{n}}\cdot J=y_{t^{\prime }}^{a}-y_{o}^{a}=y_{t}^{a}-y_{o}^{a}+\frac{\left(y_{t}^{b}-y_{t}^{a}\right)*\left(y_{t}^{a}-y_{o}^{a}\right)}{\left(b-m\right)\left(y_{t}^{a}-y_{o}^{a}\right)+\left(m-a\right)\left(y_{t}^{b}-y_{o}^{b}\right)}*\epsilon_{t}\;$$

Although the value of *m* is not known for the first iteration, it is not required, as $\epsilon_{t}$ is assumed to be 0 for the first iteration. When Equations (8) and (9) are computed for each non-origin point, the equation can be simplified to Equations (10) and (11).

(10) $$G\cdot I=x_{t^{\prime }}^{a}-x_{o}^{a}$$

(11) $$G\cdot J=y_{t^{\prime }}^{a}-y_{o}^{a}$$

where ***G*** is the relative 3D position of the non-origin points with respect to the origin point (see Equation (12)). This was found in Section 5.3.

(12) $$G=\left[\begin{array}{c} \vec{M_{o}^{m}M_{t}^{n}}\\ \vec{M_{o}^{m}M_{t1}^{n}}\\ \cdots \end{array}\right]=\left[\begin{array}{ccc} o_{1} & p_{1} & q_{1}\\ o_{2} & p_{2} & q_{2}\\ \ldots & \ldots & \ldots \end{array}\right]\;$$

Equations (10) and (11) are rearranged for ***I*** and ***J*** to give Equations (13) and (14), where ***H*** is the pseudoinverse of ***G***.

(13) $$I=H\left(x_{t^{\prime }}^{a}-x_{o}^{a}\right)$$

(14) $$J=H(y_{t^{\prime }}^{a}-y_{o}^{a})$$

To compute the rotation matrix, ***I*** and ***J*** must be converted to unit vectors ***i*** and ***j*** (see Equation (4)). ***i*** and ***j*** can be calculated by finding the scaling factor, *s*, by applying Equation (15) and dividing ***I*** and ***J*** (see Equations (16) and (17)).

(15) $$s=\left(I\cdot I\right){\;}^{0.5}=\left(J\cdot J\right){\;}^{0.5}$$

(16) i=I/s 

(17) j=J/s

Projecting $\vec{M_{o}^{m}M_{t}^{n}}$**,** or equivalently $\vec{M_{o}^{m}M_{t^{\prime }}^{m}}$, onto the unit vector ***i*** or ***j*** can be written to form Equations (18) and (19).

(18) $$\vec{M_{o}^{m}M_{t^{\prime }}^{m}}\cdot i=\left(\frac{\left(b-m\right)}{\left(b-a\right)}+\frac{\left(m-a\right)}{\left(b-a\right)}\frac{\left(x_{t}^{b}-x_{0}^{b}\right)}{\left(x_{t}^{a}-x_{0}^{a}\right)}\right)*\left(x_{t’}^{a}-x_{0}^{a}\right)=s^{-1}\left(x_{t’}^{a}-x_{0}^{a}\right)$$

(19) $$\vec{M_{o}^{m}M_{t^{\prime }}^{m}}\cdot j=\left(\frac{\left(b-m\right)}{\left(b-a\right)}+\frac{\left(m-a\right)}{\left(b-a\right)}\frac{\left(y_{t}^{b}-y_{0}^{b}\right)}{\left(y_{t}^{a}-y_{0}^{a}\right)}\right)*\left(y_{t’}^{a}-y_{0}^{a}\right)=s^{-1}\left(y_{t’}^{a}-y_{0}^{a}\right)$$

Either Equation (18) or (19) can be rearranged for *m*, giving the same value. Equation (20) is a rearrangement of Equation (18).

(20) $$m=\frac{a\left(-sx_{t}^{b}+sx_{o}^{b}+x_{t}^{a}-x_{0}^{a}\right)+b\left(s-1\right)\left(x_{t}^{a}-x_{0}^{a}\right)}{s\left(x_{t}^{a}-x_{0}^{a}-x_{t}^{b}+x_{0}^{b}\right)}\;$$

Given that ***i*** and ***j*** have been computed, ***k*** can be found by taking the cross-product of ***i*** and ***j*** (see Equation (21)).

(21) −k=i×j 

The distance $\epsilon_{t}$ can be computed by taking the dot product of $\vec{M_{o}^{m}M_{t}^{n}}$ and ***k***, as shown in Equations (22) and (23). This process can be iterated with the new values of $\epsilon_{t}$ and *m* until the values of $\epsilon_{t}$ do not change by more than a threshold value.

(22) $$n_{t}=m+\vec{M_{o}^{m}M_{t}^{n}}\cdot k=m+\epsilon_{t}\;$$

(23) $$\epsilon_{t}=\vec{M_{o}^{m}M_{t}^{n}}\cdot k$$

#### 5.5.2. Compute Position

Once $\epsilon_{t}$ has converged, the x, y, and z position of each non-origin LED can be computed using Equations (24), (25) and (26), and the rotation matrix can be computed by applying Equation (4). Note that ***x*, *y*, *****z***, and ***n*** are bold because there is more than LED.

(24) $$z=n_{t}=m+\epsilon_{t}\;$$

(25) $$y=y_{t}^{a}+\left(y_{t}^{b}-y_{t}^{a}\right)*\frac{\left(n_{t}-a\right)}{\left(b-a\right)}$$

(26) $$x=x_{t}^{a}+\left(x_{t}^{b}-x_{t}^{a}\right)*\frac{\left(n_{t}-a\right)}{\left(b-a\right)}\;$$

Note x**,** y, and z are the x, y, and z coordinates of each non-origin LED relative to the global coordinate system (i.e., the camera). The x, y, and z coordinates of the origin LED ($x_{0}$, $y_{0}$, $z_{0}$) can be found in a similar way to Equations (25) and (26) and combined in Equation (27) to form a translation matrix, T, which describes the translation from the global coordinate system to the object coordinate system.

(27) $$T=\left[\begin{array}{c} x_{0}\\ y_{0}\\ z_{0} \end{array}\right]=\left[\begin{array}{c} x_{0}^{a}+\left(x_{0}^{b}-x_{0}^{a}\right)\frac{\left(m-a\right)}{\left(b-a\right)}\\ y_{0}^{a}+\left(y_{0}^{b}-y_{0}^{a}\right)\frac{\left(m-a\right)}{\left(b-a\right)}\\ m \end{array}\right]\;$$

## 6. Optical Tracking Results

### 6.1. Camera Calibration

**Figure 7:**
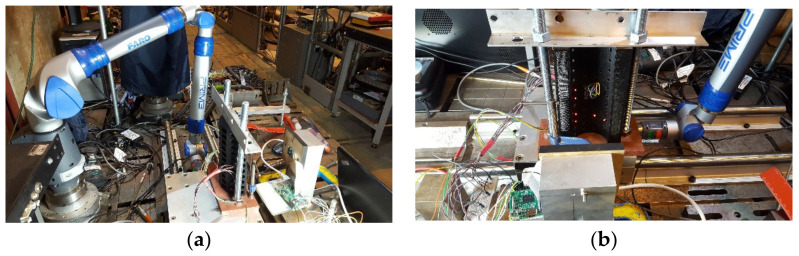
Optical tracking test setup: (**a**) Side view; (**b**) Front view.

To create a custom calibration target suitable for the application at hand, the free pattern generator services of Calib.io were used to generate a PDF template. Then, the template was converted from the default black markers on a white background to white markers on a black background to reduce the complexity of the image processing. To ensure the calibration captured the full distortion of the camera’s lens, a very dense calibration target was created with 18 × 32 2 mm diameter targets with a 4 mm spacing. Then, the target was printed using an imageRUNNER ADVANCE C5500 printer (Canon Inc., Tokyo, Japan) with a print resolution of 1200 × 1200 dpi, giving a highly precise calibration target with a maximum dimensional error of 0.01 mm. To ensure that the calibration target was not warped, the high-resolution calibration image was mounted on a 10 mm MDF board. To achieve sub-millimetre accuracy in the calibration and subsequent calculation of the brick pose, it is essential that the calibration plane is precisely aligned to the camera. As such, a calibration rig was designed using bespoke restraint components machined from aluminium and steel. The calibration setup is presented in Figure 7. Then, a FARO arm PRIME, with an accuracy of 0.027 mm and repeatability of 0.019 mm, was used to determine the position of the calibration planes from the camera. Calibration Planes A and B were prescribed at 101.117 and 161.111 mm from the camera. A fifth-order polynomial was selected, giving 21 calibration coefficients.

**Figure 8:**
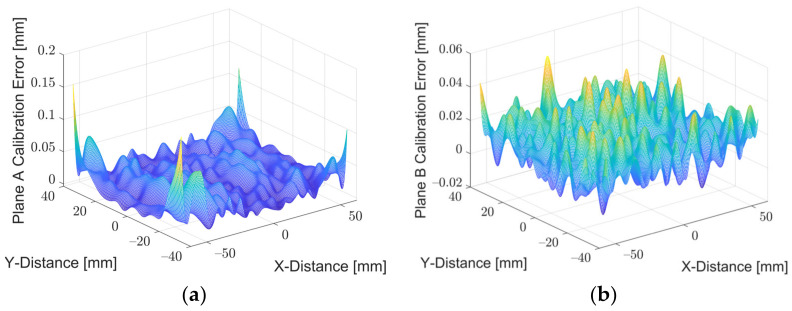
Error contours of each calibration plane: (**a**) Plane A; (**b**) Plane B.

**Figure 9:**
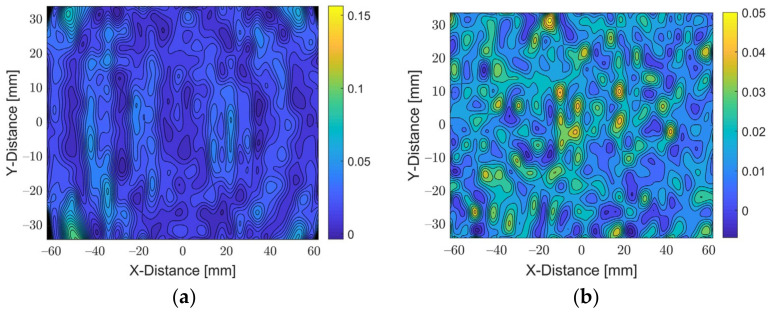
Error contours of each calibration plane: (**a**) Plane A; (**b**) Plane B.

To evaluate the performance of the calibration, the coefficients were reapplied to the u and v calibration points found at Planes A and B. The error could be computed by comparing the position given by the calibration and the known position. The average and maximum error for Plane A were calculated to be 23.7 µm and 161.8 µm, respectively. Meanwhile, for Plane B, the average and maximum error were calculated to be 16.1 µm and 46.1 µm, respectively. The errors are visualised in Figure 8 and Figure 9 by a 3D error distribution map and an error contour. The error is small and noisy across both planes, only deteriorating in small areas at corners of the image, indicating that the calibration is robust and effective. This error could be further reduced by increasing the number of markers at the extremities of the frame, allowing the IRT calibration to account for the lens distortion and further improve the accuracy of the results.

### 6.2. PRSOI Accuracy

**Figure 10:**
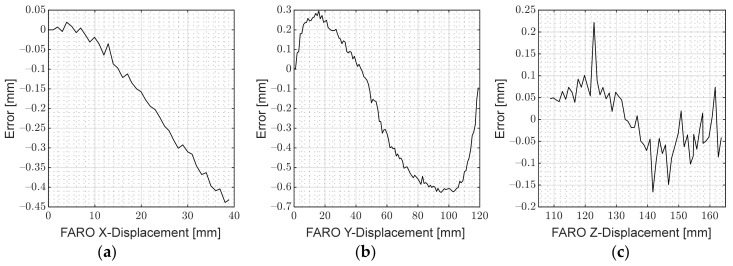
PRSOI-IRT: (**a**) X-error; (**b**) Y-error; (**c**) Z-error.

To find the z-error of the system, the brick was clamped to the translation plate, and the FARO arm was connected. Then, the plate was translated between Plane A and B, taking a FARO reading and image for each millimetre. The optical algorithm was applied to each image, giving out a positional reading, which was compared to the FARO arm reading to give an error value, as shown in Figure 10c. The system was found to have a maximum z-error of 0.2220 mm and RMS error of 0.0703 mm. The flatness of the error suggests that the translation plate has been aligned precisely and accurately, while the low noise suggests that the image processing is robust and accurate in finding the centre of mass of the LEDs.

For the x- and y-axis error determination, both the optical algorithm and FARO arm were zeroed at one corner of the field of view (FOV) of the camera; then, they were translated in 1 mm increments until the brick reached the opposite corner of the FOV. The x- and y-axis error are shown in Figure 10a and b, respectively. The maximum and RMS error was 0.4386 mm and 0.2296 mm for x, and 0.6262 mmm and 0.3943 mm for y. The x-axis error shows a linear trend, which is likely due to misalignment of the camera and translation table. The y-axis error has a systematic sine-like error, which may be due to the 3D hemisphere centre of mass of the LEDs not being exactly the same as the 2D centre of mass. Nevertheless, the errors and noise are small, testifying to a rigorous and accurate image filtering methodology, a robust correspondence algorithm, and precise calibration.

Although the angular precision of the algorithm could not be tested due to a lack of instrumentation, which could provide accurate angular manipulation, it was assumed the angle of the clamped brick did not change appreciably, as the brick was translated in the x-, y-, and z-direction. The angle of rotation about each axis was computed by converting the rotation matrix into Euler angles. The maximum difference in the Euler x, y, and z angles for the x-translation were found to be 1.21°, 0.46°, and 0.37°; for the y-translation, they were 1.54°, 2.88°, and 0.40°; and for the z-translation, they were 0.54°, 0.38°, and 0.34°. These results are similar to the maximum errors reported by Sun et al. [21] and Guo et al. [23], who implemented the same algorithm. This suggests that the instrumentation, as reported by Sun et al. [21] and Guo et al. [23], produces sub-degree accurate results. However, this should be investigated empirically to validate this result. As the optical tracking system is shown to be highly accurate for each of the x, y, and z components, the optical tracking can be implemented to correct the drift of the IMU.

The error of the system described in this paper is almost an order of magnitude smaller than that produced by deep learning optical algorithms, which are accurate to ≈3 mm and ≈3° [20,24]. Similar sub-millimetre levels of accuracy in optical tracking systems have been achieved in few other systems e.g., Tiaden et al. [8].

The computational efficiency of the code was determined by running the PRSOI-IRT algorithm for 50 frames. The code took 137.66 s to run and gave the computational efficiency of 2.75 s for each frame on a Lenovo Thinkpad E470. As the camera is expected to run for 15 s at 30 FPS, roughly 450 frames will be captured, which equates to 21 min of processing time. Although large, the post-processing time of the data is not a priority, and 21 min is deemed to be suitable to produce sub-millimetre and sub-degree accuracy results. Future work could focus on multithreading the algorithm to reduce this time significantly.

## 7. Timestamping and Aligning the Axes of the Optical and Inertial Data

To fuse the optical and IMU systems, the data collected from both systems must be timestamped correctly. To satisfy this requirement, both the IMU and camera must start collecting data at a known time and proceed to accurately timestamp the data after initialisation.

### 7.1. Optical Tracking Timestamps

To test the accuracy of the timestamps recorded by the camera, MATLAB’s free psychtoolbox-3 was used. This toolbox interfaces between MATLAB and computer hardware to produce sub-millisecond accuracy displays. The psychotoolbox accurate timing demo was used to rapidly output different coloured frames as the laptop’s screen refreshed. These displays were captured by the camera and used as a timing reference for the timestamps.

#### 7.1.1. Screen Synchronisation

To effectively implement the MATLAB psychotoolbox, the computer screen must be shown to output coloured screens at the correct time. The refresh rate of the screen was tested using two independent methods: the beam position interval and the Vertical Blanking Interval (VBI).

A computer screen is refreshed in a similar manner to the way a rolling camera takes a photo, with each line of the screen being refreshed, one at a time, by an electron beam in a Cathode Ray Tube (CRT) or by applying a voltage in a Liquid Crystal Display (LCD). Once the refreshing “beam” has reached the end of a row, there is a brief pause known as the horizontal blanking interval, which allows the beam to move to the next row. Once the beam has finished the last row of the screen, a comparatively longer pause, known as the VBI, allows the beam to move back to the top of the screen. Timestamps are taken at the start of the VBI and when the beam has returned to a selected position on the screen.

**Figure 11:**
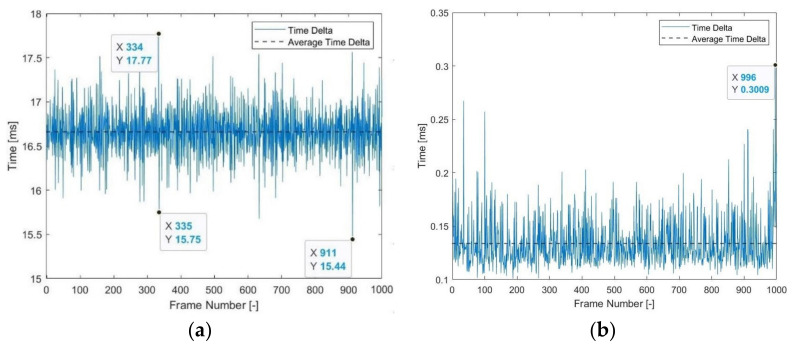
Screen synchronisation: (**a**) Delta between successive refreshes according to beam position timestamp; (**b**) Time difference between requested and returned VBI timestamp.

To test that the screen outputs the colours at the correct time, the screen is refreshed 1000 times at 60 Hz (refresh every 16.67 ms), collecting a timestamp when the beam reaches a specified position and the VBI timestamp with each refresh. The beam position timestamp demonstrated that the screen, on average, has an error of 0.0055 ms (0.03%) and a maximum error of 1.2260 ms (7.36%), as shown in Figure 11a. The VBI timestamp gave an average error of 0.0169 ms (0.10%) and a maximum error of 0.1672 ms (1.00%), as presented in Figure 11b. The error in both responses is due to unavoidable jitter and drift in the laptop’s Nvidia GeForce 940MX graphics card (Nvidia Corporation, Santa Clara, CA, United States) which is caused by the operating temperature of the laptop, fluctuations in the power supply, and other instabilities. Nonetheless, the maximum and average percentage error are small with respect to the exposure of the camera and, therefore, the screen synchronisation can be assumed to output the coloured screens at the correct time.

#### 7.1.2. Optical Tracking Timestamps

For the camera to produce a similar frequency to that of the computer screen (60 FPS), the resolution of the camera was reduced to 640 × 480 pixels. This assumes that if the camera accurately timestamps at 60 Hz, the camera will also accurately timestamp at lower frequencies and higher resolutions. The timestamps were captured using the presentation timestamp. This is the time at which the Raspberry Pi receives the first line of the frame data. The value returned is given by the encoder in microseconds since the video started recording, preventing timestamp inaccuracies due to limited computational resources.

**Figure 12:**
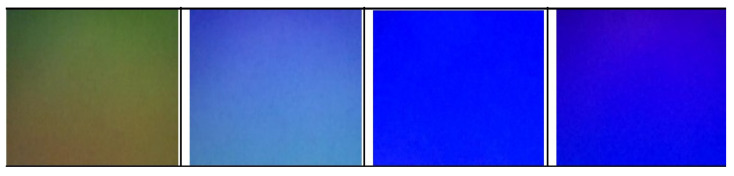
Images captured when the timestamps have an error of half a frame.

To check whether the timestamps are accurate, the screen was programmed to output a green, purple, blue, and red screen in succession. Using the timestamps of the images, the drift of the image capture with respect to the screen can be computed. Images can be compared when the timestamps report they have drifted exactly an integer number of frames. If the timestamps are accurate, the frames should maintain their colour but be displaced one frame backward for each frame of drift. For example, one frame of drift would yield a result of green becoming red or purple becoming green. If the camera images drift half a frame (8.33 ms) with respect to the screen, then the images will not maintain their colour but produce images with modified colours, such as the results shown in Figure 12.

**Figure 13:**
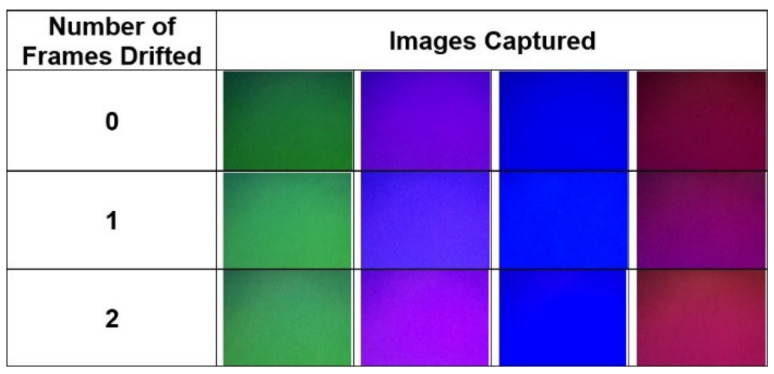
Images captured when the timestamps have reported drifting an integer number of frames. Note that the relevant colours have been re-aligned as a visual aid to ease comparison of colours.

To ensure that the timestamp accuracy could be maintained for the typical 15 s duration of an MLA test, the screen and camera were tested for a 20 s period. In this time, the camera reported running at 60.2 Hz and drifting two full frames with respect to the screen. The initial images captured were compared with their corresponding colours at one and two frames of drift from 60 Hz, as shown in Figure 13. The frames with a reported drift of one and two frames yielded screen colours that were more similar to the initial screen colours than those exhibited when there is 8.33 ms of error as presented in Figure 12, showing that the error was less than 8.33 ms. As such, the timestamps were considered fit for purpose. Note that as an image was taken every 16.67 ms, these images presented are only a small subset of the images captured during the validation of the timestamps for conciseness, and the images taken were verified to drift only one frame rather than multiple frames.

#### 7.1.3. Camera Initialisation Time

**Figure 14:**
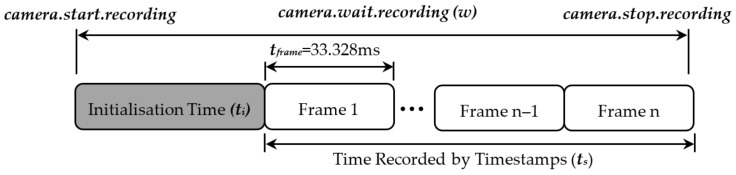
Optical tracking initialisation time.

To investigate the initialisation time of the camera, the camera was run for a fixed time, *w*. The number of frames captured during this time, $n_{frames}$, and their corresponding timestamp can be subtracted from *w* to calculate the initialisation time, *t_i_* (see Figure 14). However, this does not give a precise value for *t_i_* because when the camera receives the signal to stop recording, it will finish capturing the current frame. Hence, the time at which the recording is stopped could be anywhere between the start and end of the last frame. Therefore, the data must be binned into frames, taking each bin’s time value as the time halfway through the last captured frame, *n* (see Equation (28)). If *w* is reduced so that it falls exactly between two frames, the variability in *t_i_* will cause equal proportions of *n* and *n −* 1 frames to be captured. By running the camera multiple times, the variability in *t_i_* can be accounted for by taking the average of the discrete initialisation times (Equation (29)). Note that as *w* is fixed and $\left(n_{frames}-\frac{1}{2}\right)t_{frame}$ has discrete values based on the variability of $t_{i}$, $t_{i}$ will be discrete.

(28) $$t_{i}=w-\left(n_{frames}-\frac{1}{2}\right)t_{frame}\;$$

**Figure 15:**
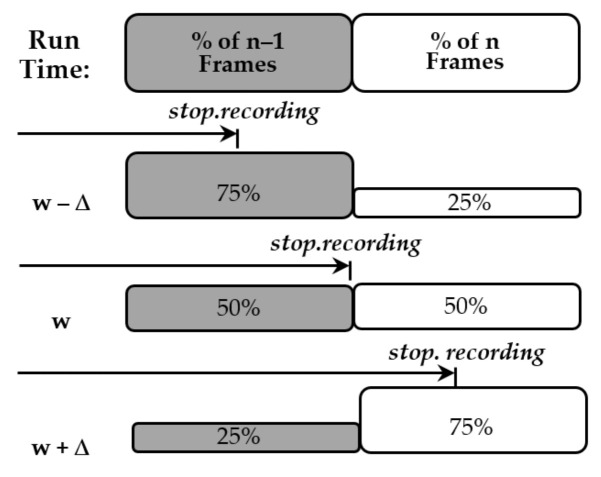
Histograms showing the effect of incrementing the wait/stop time.

Further data and a more accurate value of *t_i_* can be computed by incrementing *w*. Increasing *w* will increase the proportion of *n* frames and reduce the proportion of *n*-1 frames, and decreasing *w* will do the opposite (see Figure 15). Changing *w* will result in an estimate of the same average initialisation time. Averaging these estimates will reduce inaccuracies the calculated initialisation time. The discrete average of the data can be taken by applying Equation (29). The standard deviation of the initialisation time can be computed by applying Equation (30). When calculating the standard deviation Sheppard’s correction factor, $t_{frame}^{2}$/12, is applied, as using the midpoint of observed binned data results in an overestimate of the standard deviation [25].

(29) $$\bar{t}=\frac{1}{N}\overset{N}{\underset{f=1}{\sum }}t_{i,f}x_{f}$$

(30) $$\sigma_{corrected}=\sqrt{\frac{\sum_{f=1}^{N}\left(t_{i,f}{}^{2}x_{f}\right)-N{\bar{t}}^{2}}{N-1}-\frac{t_{frame}^{2}}{12}}\;$$

The variable $x_{f}$ is the number of times a particular discrete initialisation time *t_i_*_,_ *_f_* was outputted, *N* is the total number of runs undertaken (or equivalently the sum of $x_{f}$), $\sigma_{corrected}$ is the corrected standard deviation, and $t_{frame}$ is the time taken to capture one frame according to the timestamps. The timestamps can be considered to be accurate and not to drift, as shown in Section 7.1.2.

To test the initialisation time, the camera was run for 3.3328 s, which is equivalent to 100 frames at a requested 30 FPS according to the presentation timestamps (as it is known that a setting of 30 FPS on the camera in reality yields an FPS of 30.005, which must be accounted for in this case). When ran for 3.3328 s, the camera was found to consistently produce 92 frames. The camera was found to produce roughly equal proportions of 91 and 92 frames when run for 3.3035 s. Then, the camera was tested between durations of 3.3005 and 3.3060 s, in increments of 0.5 ms with 100 iterations for each duration.

The initialisation time was found to have a standard deviation and a mean value of 0.01686 s and 0.2857 s, respectively. As the standard deviation is small, the average initialisation time can be added to each frame’s timestamps to generate timestamp values that are relative to the input trigger signal. Although the standard deviation is low, 2.38% of the 1100 data values are outside 5% of the average with large changes in the initialisation time. This could be due to the Raspberry Pi scheduling other tasks, resulting in a delay in the image capture; however, the percentage of such instances are small. Further investigation of the root cause of this issue was not attempted in this work, because when the hardware is miniaturised, by removing all non-essential ports and chips from a standard Raspberry Pi, the problem may disappear naturally. However, given this is currently an issue, the IMU and optical data can be time-synchronised during post-processing by aligning the motion of the two tracking systems in time.

### 7.2. IMU Timestamps

**Figure 16:**
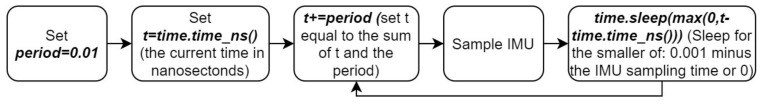
IMU time synchronisation algorithm.

The timestamps of the IMU must also be shown to not drift, and the initialisation time must be quantified. The IMU initialisation is given as 400 ms by the manufacturer and can be taken into account in the sensor fusion. Although the IMU data will not drift, as the BNO055 has an internal 32 kHz clock source, the sampling of this data by the Raspberry Pi may drift. To ensure that the sampling of the IMU is at 100Hz and drift free, an adaptive time.sleep function was implemented, as shown in Figure 16. This code will not drift if the sampling of the IMU takes less than 0.01 s. To test this, the code was run for 10,000 iterations. The maximum sample time was found to be 0.00853 s, and therefore, it can be assumed that the IMU can be sampled at 100 Hz with no drift over time.

### 7.3. Common Coordinate System between Optical and IMU Data

To fuse the optical and inertial coordinate systems, the rotation and translation matrix between the two coordinate systems must be identified. As the PLA brick was printed with a small flat surface at the centre of the brick, which is parallel with the six coplanar LEDs and the o, p axis of the object coordinates system, the rotation matrix is an identity matrix without rotation. The translation between the IMU and origin LED was computed using the in-built functionality of Autodesk Inventor, giving a translation matrix, in mm, of [26.045, 53.000, 14.199] in [x, y, z]. Then, the BNO055 axis was re-referenced using the AXIS_MAP_CONFIG register, allowing the IMU data to be expressed in the same coordinates as the optical system.

## 8. System Development

Given that the data of both systems are timestamped and in the same coordinate system, they can be sensor-fused using an Extended Kalman Filter (EKF). EKFs have shown to fuse systems to produce more accurate data than either single system and at the sampling rate of the faster system (IMU in this case). Rambach et al. [9] implements an EKF for a Head-Mounted Display (HMD), which fuses optical and IMU signals in a system similar to the one outlined in this paper. The EKF can be implemented in the current system, removing the constant linear and angular velocity forward prediction, since all data outputs are post-processed, which is likely to improve the results further.

As the system has been shown to accurately find the position of the brick, while satisfying all problem constraints, future work will focus on developing the hardware so that it can be implemented in the MLA rig. In particular, the system will be implemented on a QCB rather than a DCB, be miniaturised to fit in the MLA, and use a camera with a smaller focal length to focus on all LEDs on the opposing brick component in the MLA.

The hardware could be miniaturised by creating a custom-made Raspberry Pi, removing all non-essential ports and chips. When miniaturising the hardware, the method of data transfer should be considered. Although Ethernet and Wi-Fi could be used on the current board, these chips are large, expensive, and it can be complex to send multiple signals to a single, external computer. A CAN bus could be favourable, as it allows multiple devices to communicate and coordinate the transfer of data along one long wire, it is low cost, and the chips tend to be small and light. Once the hardware is miniaturised, to set up each brick before being placed in the MLA, the camera calibration of each MLA brick must take two pictures of the calibration target at two selected z-plane positions.

Future work will also focus on the improving the robustness of the system by investigating two issues: (1) Compensating for LED’s potentially leaving the FOV of the camera and (2) reducing motion blur. The effects of motion blur have been reduced successfully in a number of studies. In particular, Savkin et al. [10] have reported an effective method for correctly finding the centre of mass of multiple LEDs based on the geometry of the motion blur and IMU acceleration data.

To ensure that the system can account for the loss of an LED, a more sophisticated correspondence algorithm is required to identify the missing LED. This will be incorporated through the development of the correspondence algorithm or the use of a well-researched algorithm such as softassign [17]. This would ensure that the optical tracking will always be able to output optical tracking data, but the effect on the accuracy of the algorithm needs to be investigated.

## 9. Conclusions

This paper presents a sensor fusion methodology that combines a low-cost optical and inertial measurement system to produce dynamic measurements of cracked bricks in a model of an AGR core, contributing towards the life extension of UK nuclear power generation. The optical system tracks LED indicators in video frames, allowing points of reference on the brick to be located in cluttered images. The indicators are localised through a robust and rigorous image filtering process and identified through a computationally efficient correspondence algorithm. The methodology is highly versatile and can be applied to any monocular camera due to a precise black-box IRT calibration that corrects complex distortion. The calibration was found to have a mean error of 0.0349 mm across the entire plane.

The six DOF pose was calculated using a PRSOI algorithm. The optical methodology was shown to be highly effective, giving an RMS error of 0.2296, 0.3943, and 0.0703 mm in x, y, and z with low noise. This high accuracy allows the optical tracking system to reliably correct the drift of an IMU. The paper also describes a methodology to align the optical and IMU data in time and space by synchronising the two motion-tracking coordinate systems and timestamping the data. Future work involves miniaturisation of the optical and inertial tracking system and fusion via a tightly coupled EKF. This cheap but compact instrumentation system will allow monitoring of the relative motions of the TCB and QCB bricks during future testing of the MLA, which is something that has not been possible to date. To monitor a TCB, two camera systems will be installed on one component with the corresponding LEDS in the other two components. This will allow relative monitoring of all three components. To monitor a QCB, three systems will be used. This system could also be used where any accurate but lightweight tracking system is needed, in particular where there is a need to use wide-angle lenses, as the item to be tracked is close to the reference point.

## Data Availability

The data presented in this study are available on request from the first author.

## References

[1] Department for Business, Energy & Industrial Strategy and Nuclear Decommissioning Authority (2019). Advanced Gas-Cooled Reactor (AGR) Decommissioning.

[2] Steer A.G., Neighbour G.B. (2007). AGR Core Design, Operation and Safety Functions. Management of Ageing in Graphite Reactor Cores.

[3] Dihoru L., Oddbjornsson O., Kloukinas P., Dietz M., Horseman T., Voyagaki E., Crewe A.J., Taylor C.A., Steer A.G. (2017). The development of a physical model of an Advanced Gas Cooled Reactor core: Outline of the feasibility study. Nucl. Eng. Des..

[4] Dihoru L., Crewe A.J., Horseman T., Dietz M., Oddbjornsson O., Kloukinas P., Taylor C.A. (2019). A Computer Vision Approach for Dynamic Tracking of Components in a Nuclear Reactor Core Model. Nucl. Eng. Des..

[5] Oddbjornsson O., Kloukinas P., Gokce T., Bourne K., Horseman T.R., Dihoru L., Dietz M., White R.E., Crewe A.J., Taylor C.A. (2021). Design and Calibration of a Hall Effect System for Measurement of Six Degree-of-Freedom Motion within a Stacked Column. Sensors.

[6] Dihoru L., Oddbjornsson O., Crewe A.J., Taylor C.A. (2021). Measurement techniques for column interface monitoring in an advanced gas cooled reactor model. Nucl. Eng. Des..

[7] He C., Kazanzides P., Sen H.T., Kim S., Liu Y. (2015). An inertial and optical sensor fusion approach for six degree-of-freedom pose estimation. Sensors.

[8] Tjaden H., Schwanecke U., Stein F., Schömer E. High-Speed and Robust Monocular Tracking. Proceedings of the 10th International Conference on Computer Vision Theory and Applications.

[9] Rambach J., Pagani A., Lampe S., Reiser R., Pancholi M., Stricker D. Fusion of Unsynchronized Optical Tracker and Inertial Sensor in EKF Framework for In-car Augmented Reality Delay Reduction. Proceedings of the 2017 IEEE International Symposium on Mixed and Augmented Reality, ISMAR-Adjunct 2017.

[10] Savkin P.A., Saito S., Vansteenberge J., Fukusato T., Wilson L., Morishima S. Outside-in monocular IR camera based HMD pose estimation via geometric optimization. Proceedings of the ACM Symposium on Virtual Reality Software and Technology, VRST.

[11] Fischler M.A., Bolles R.C. (1981). Random sample consensus: A Paradigm for Model Fitting with Applications to Image Analysis and Automated Cartography. Commun. ACM.

[12] Dementhon D.F., Davis L.S. (1995). Model-based object pose in 25 lines of code. Int. J. Comput. Vis..

[13] Kneip L., Li H., Seo Y., Fleet D., Pajdla T., Schiele B., Tuytelaars T. (2014). UPnP: An Optimal O(n) Solution to the Absolute Pose Problem with Universal Applicability. Computer Vision–ECCV 2014.

[14] Lu C.P., Hager G.D., Mjolsness E. (2000). Fast and globally convergent pose estimation from video images. IEEE Trans. Pattern Anal. Mach. Intell..

[15] Urban S., Leitloff J., Hinz S. (2016). MLPnP-A Real-Time Maximum Likelihood Solution to the Perspective-n-Point Problem. ISPRS Ann. Photogramm. Remote Sens. Spat. Inf. Sci..

[16] David P., Dementhon D., Duraiswami R., Samet H. (2003). SoftPOSIT: Simultaneous pose and correspondence determination. Int. J. Comput. Vis..

[17] Rangarajan A., Chui H., Bookstein F.L. (1997). The softassign procrustes matching algorithm. Lect. Notes Comput. Sci. (Incl. Subser. Lect. Notes Artif. Intell. Lect. Notes Bioinform.).

[18] Zheng Q., Zhao P., Zhang D., Wang H. (2021). MR-DCAE: Manifold regularization-based deep convolutional autoencoder for unauthorized broadcasting identification. Int. J. Intell. Syst..

[19] Carrillo L.R.G., Vamvoudakis K.G. (2020). Deep-Learning Tracking for Autonomous Flying Systems under Adversarial Inputs. IEEE Trans. Aerosp. Electron. Syst..

[20] Garon M., Lalonde J. (2017). Deep 6-DOF Tracking. IEEE Trans. Vis. Comput. Graph..

[21] Sun P., Sun C., Li W., Wang P. (2015). A new pose estimation algorithm using a perspective-ray-based scaled orthographic projection with iteration. PLoS ONE.

[22] Kyriakoulis N., Gasteratos A. (2010). Color-Based Monocular Visuoinertial 3-D Pose Estimation of a Volant Robot. IEEE Trans. Instrum. Meas..

[23] Guo X., Tang J., Li J., Shen C., Liu J. (2019). Attitude measurement based on imaging ray tracking model and orthographic projection with iteration algorithm. ISA Trans..

[24] Deng H.W., Zhu W. Monocular Free-Head 3D Gaze Tracking with Deep Learning and Geometry Constraints. Proceedings of the 2017 IEEE International Conference on Computer Vision (ICCV).

[25] Hooda R.P. (2013). Statistics for Business and Economics.

